# Draft Genome Sequence of *Bacillus cereus* 905, a Plant Growth-Promoting Rhizobacterium of Wheat

**DOI:** 10.1128/genomeA.00489-16

**Published:** 2016-06-02

**Authors:** Haixia Ding, Ben Niu, Haiyan Fan, Yan Li, Qi Wang

**Affiliations:** Key Laboratory of Plant Pathology, College of Plant Protection, China Agricultural University, Beijing, China

## Abstract

*Bacillus cereus* 905 is a plant growth-promoting rhizobacterium, isolated from wheat rhizosphere. The draft genome sequence of this strain is 5.39 Mb and harbors 5,412 coding sequences.

## GENOME ANNOUNCEMENT

Plant growth-promoting rhizobacteria (PGPR) have been applied as environmentally friendly alternatives to agrochemicals to improve crop yields and quality (1). *Bacillus* is a well-studied genus containing a substantial number of PGPR strains belonging to *B. cereus*, *B. subtilis*, *B. amyloliquefaciens* (2, 3) and so on, which are capable of promoting plant growth and suppressing phytopathogens (4, 5). *B. cereus* 905, isolated from wheat rhizosphere, exhibited significant plant growth-promoting effects (6). One commercial product developed from 905 has been applied to wheat fields of approximately 3 million acres after being registered as a biopesticide. Although the results from a previous study show that 905 is an efficient colonizer of wheat roots and that two manganese superoxide dismutases are essential in colonization (6), our knowledge about the mechanisms of 905’s plant growth-enhancing activity is still very limited. In this work, we report the draft genome sequence of 905, which will serve as a useful genetic reference for the investigation of *B. cereus*–plant interaction.

The draft genome sequence of 905 was determined by next-generation sequencing technology (7). The genomic DNA of 905 was used for construction of a 3-kb-long paired-end library with a GS FLX library preparation kit in combination with GS FLX paired-end adaptors (both from Roche, Mannheim, Germany) according to the manufacturer’s protocol. The sequencing was performed on the Genome Sequencer FLX platform (Roche, Mannheim, Germany). The output reads were then assembled using the GS *De Novo* Assembler software program. In total, 1,057,588 reads, including 436,109 paired reads, were assembled with a total of 179,419,730 bp. Utilization of the paired-end information allowed scaffolding of the contigs larger than 500 bp. Gap closure was partially done by long-range PCR (using Phusion polymerase; New England BioLabs, Frankfurt am Main, Germany) and subsequent Sanger sequencing (IIT Biotech, Bielefeld, Germany). In total, the genome assembly yielded 126 scaffolds with an *N*_50_ scaffold size of 91,419 bp.

The draft genome sequence of 905 consists of 5,386,583 bp, with a G+C content of 35%. A total of 5,412 coding sequences were identified in the genome, a considerable number of which were predicted and annotated as genes involved in bacterium–plant interaction and biocontrol of phytopathogens, such as the genes related to chemotaxis, sporulation, biofilm formation, biosynthesis of siderophore, bacteriocin, polyketide, protease, superoxide dismutase, endoglucanase, cellulase, chitinase, and so on. The draft genome sequence reported here gives us an overview of the PGPR features of 905 and provides valuable genetic information that will facilitate further study on how 905 interacts with plants.

### Nucleotide sequence accession numbers.

This whole-genome shotgun project has been deposited in DDBJ/ENA/GenBank under the accession number LSTW00000000. The version described in this paper is the first version, LSTW01000000.
